# Induced mesenchymal stem cells generated from periodontal ligament fibroblast for regenerative therapy

**DOI:** 10.3389/ebm.2025.10342

**Published:** 2025-02-03

**Authors:** Hemanathan Vembuli, Sheeja Rajasingh, Patrick Nabholz, Jefferson Guenther, Brian R. Morrow, Margaret M. Taylor, Marziyeh Aghazadeh, Vinoth Sigamani, Johnson Rajasingh

**Affiliations:** ^1^ Department of Bioscience Research, University of Tennessee Health Science Center, Memphis, TN, United States; ^2^ Department of Medicine-Cardiology, University of Tennessee Health Science Center, Memphis, TN, United States; ^3^ Department of Microbiology, Immunology and Biochemistry, University of Tennessee Health Science Center, Memphis, TN, United States

**Keywords:** mesenchymal stem cells, induced pluripotent stem cells, differentiation, osteocytes, regenerative therapy

## Abstract

Bone fractures and bone loss represent significant global health challenges, with their incidence rising due to an aging population. Despite autologous bone grafts remain the gold standard for treatment, challenges such as limited bone availability, immune reactions, and the risk of infectious disease transmission have driven the search for alternative cell-based therapies for bone regeneration. Stem cells derived from oral tissues and umbilical cord mesenchymal stem cells (MSCs) have shown potential in both preclinical and clinical studies for bone tissue regeneration. However, their limited differentiation capacity and wound healing abilities necessitate the exploration of alternative cell sources. In this study, we generated induced pluripotent stem cells (iPSCs) using a safe, nonviral and mRNA-based approach from human periodontal ligament fibroblasts (PDLF), an easily accessible cell source. These iPSCs were subsequently differentiated into MSCs, referred to as induced MSCs (iMSCs). The resulting iMSCs were homogeneous, highly proliferative, and possessed anti-inflammatory properties, suggesting their potential as a superior alternative to traditional MSCs for regenerative therapy. These iMSCs demonstrated trilineage differentiation potential, giving rise to osteocytes, chondrocytes, and adipocytes. The iMSC-derived osteocytes (iOSTs) were homogeneous, patient-specific and showed excellent attachment and growth on commercial collagen-based membranes, highlighting their suitability for bone tissue regeneration applications. Given their promising characteristics compared to traditional MSCs, PDLF-derived iMSCs are strong candidates for future clinical studies in bone regeneration and other regenerative dental therapies.

## Impact statement

We introduced a new and easily accessible homogeneous induced mesenchymal stem cells from dental tissue, which can be readily obtained during routine tooth extractions or surgeries for personalized regenerative dentistry. These stem cells represent a unique alternative form of stem cells distinct from traditional MSCs, with better proliferation activity, immunomodulatory function, and wound-healing properties, making them a promising candidate for advancing regenerative dental therapies.

## Introduction

Bone fractures and bone loss are significant health‏ problems worldwide, and their incidence is increasing due to the aging population [1]. These problems arise from the slowing down of the bone remodeling process and the reduction of minerals in the extracellular matrix. Congenital abnormalities, trauma, infectious diseases and cancer contribute to the growing number of patients needing bone reconstruction [2, 3]. Despite autologous bone grafts being considered the gold standard, surgeons and patients face various challenges with current treatments. These include the shortage of bone sources, immune reactions, transmission of infectious diseases, and difficulties in graft harvesting [4, 5]. These challenges have limited the development of cell-based therapies for bone regeneration. Dental-derived stem cells, obtained from extracted, impacted, and exfoliated teeth, are a promising source for regenerative medicine due to their availability and potential for multilineage differentiation. Numerous preclinical studies have demonstrated their potential in treating medical conditions including ischemic disease [6, 7], neural tissue injuries [8, 9], diabetes [10, 11], skin and hair injuries [12, 13], muscular dystrophy [14–16] and cartilage defects [17, 18]. Dental-derived cells have also been successfully used for bone tissue regeneration in several preclinical and clinical studies [19–23].

Umbilical cord mesenchymal stem cells (UC-MSCs) are another source of multilineage stem cells with properties such as high self-renewal and low immunogenicity. They are isolated and cultured from umbilical cord [24]. For years, the storage of UC-MSCs has been utilized worldwide as a source of cells for future therapy. In some countries, at the time of delivery, most parents are advised to preserve the umbilical cord stem cells or umbilical blood in a stem cell bank for potential use in autologous stem cell therapy. However, this option is only available at the time of birth and is very expensive, making it impractical for the majority of people [25]. Mesenchymal stem cells (MSCs) from bone marrow, adipose tissues and umbilical cord blood have been shown to be promising alternative sources for MSCs and have become important in regenerative medicine [26–29]. However, these cells from human body are not easily accessible due to invasive and discomforting procedures. Moreover, these cells are categorized as adult stem cells, and their differentiation capacity is limited compared to embryonic stem cells. Access to embryonic stem cells is not feasible, so scientists are striving to develop an alternative method to generate induced pluripotent stem cells (iPSCs), a promising cell sources for treating human diseases.

This iPSC technology provides a basic platform for generating patient-specific pluripotent stem cells and subsequently differentiating them into specific cell lineages for cell therapy due to their remarkable proliferation ability and immune compatibility. However, the reprogramming approach for deriving iPSCs needs improvement in terms of technique, efficiency, and availability of primary autologous cell sources. Initially, these cells were developed using retro- and lentiviruses to deliver vectors containing genes required for cell reprogramming. One major disadvantage of this method is the uncontrolled integration of viral DNA into host cell genomes [30]. To address this limitation, different methods have been developed. Our non-viral and mRNA-based reprogramming method, utilizing mRNA of pluripotent genes and a cocktail of miRNAs, has demonstrated high efficiency and the production of high-quality autologous iPSCs with high replicative and differentiation potentials, suitable for regenerative therapy [31]. These iPSCs present new opportunities for understanding embryogenesis and a great impact on drug screening and toxicological tests [26].

Human periodontal ligament fibroblasts (PDLF) are easily accessible cells that can be obtained from extracted teeth. In this study, we utilized PDLF as an optimal source for generating iMSCs which is similar to UC-MSCs and evaluated the efficacy of PDLF derived iMSCs in regenerative therapy.

## Materials and methods

### Antibodies and reagents

Primary antibodies for OCT4, NANOG, SOX2 (Cell Signaling Technology), β-actin, TRA1-60, SSEA4, VE-Cadherin, α-fetoprotein (AFP) (Santa Cruz Biotechnology, Inc.) and Nestin (R&D Systems) were used to perform *in vitro* protein analysis (Supplementary Table S1). Secondary antibodies APC. TRITC, PE and FITC-conjugated anti-donkey, anti-mouse, anti-goat and anti-rabbit (Jackson ImmunoResearch Laboratories, Inc.) were used respectively. Culture media including NutriStem (NS) medium, Mesencult medium (Stem Cells), reprogramming kits (Repro cell) and DAPI stain (Life Technologies) were used in this study. RNA of cells was collected using TRIzol reagent (Ambion by Life Technologies), and the quantification of the samples was evaluated by the NanoDrop 8000 Spectrophotometer (Thermo Fisher). The list of the primers for specific gene expression is mentioned in Supplementary Table S2. The isolated protein samples were quantified by Bradford’s method using the AccurisTM instrument SmartReader and the absorbance read at 595 nm.

### PDLF and UC-MSC cell culture

Human PDLF cell line from Lonza (Walkersville, MD; cc-7049) was gifted by Dr. Ammaar Abidi. Cells were grown in a 60-mm or 100-mm dish containing SCBM fibroblast culture medium (Lonza, United States) supplemented with 15% FBS, and 1% penicillin/streptomycin antibiotics and maintained at 37°C in a 5% CO_2_ incubator. The human UC-MSCs (referred to as MSCs) were purchased from ScienCell Research Laboratories. The cells were cultured in Mesencult medium (Reprocell, United States) and maintained as per supplier’s instruction. When the cells become 80% confluent, they were sub-cultured and used for reprogramming experiments.

### Non-viral reprogramming of human PDLF cell-derived iPSCs (referred to as iPSCs)

PDLF cells were sub-cultured in NS medium (Stemcell Technologies, Canada) in a 6-well plate coated with iMatrix (Reprocell USA Inc). At 80% confluency, the cells were reprogrammed with the mRNA of OCT4, NANOG, SOX2, KLF4, MYC and LIN28 along with a cocktail of microRNAs (Reprocell USA Inc) using Lipofectamine RNA iMAX transfection agent [32, 33]. After 8 days, several iPSC-granulated colonies were generated which resembled human embryonic stem cell colonies. These colonies were characterized for pluripotency using real time quantitative PCR, Western blot, and immunofluorescence analyses. TRA1-60, a marker of pluripotent stem cells, was employed to identify positive colonies. These colonies were manually picked from day thirteen onwards and subsequently cultured and maintained on Matrigel-coated plates in NS medium.

### Trilineage differentiation of iPSCs

To evaluate the trilineage differentiation potential of iPSCs, we differentiated them into three distinct cell lineages: mesoderm, endoderm, and ectoderm [32].

For mesoderm differentiation, specifically endothelial cells (ECs), iPSCs were cultured in NS medium in a 30-mm culture dish. When the cells reached 70%–80% confluency, the NS medium was replaced with mesodermal medium (DMEM supplemented with 1X B27, 1X N2, 5 μM CHIR, 25 ng BMP4) and cultured for 3 days. After 3 days, mesodermal medium was replaced with StemPro34 medium for 4 days, followed by the addition of endothelial EGM2 medium to promote the development of matured ECs. The generated ECs were characterized for endothelial-specific genes VE-cadherin, and CD31by qRT-PCR analysis and protein expressions by immunofluorescence analysis.

For endoderm differentiation, iPSCs were cultured in NS medium. When the iPSCs reached 70%–80% confluency, cells were cultured with Stemdiff definitive endoderm medium (Stemcell Technologies). Twelve days post culture, we observed that these cells exhibited a cuboid shaped morphology representing primary hepatocytes. The cells were harvested and subjected to qRT-PCR analysis to evaluate the gene expression of hepatocyte specific markers, including apolipoprotein A1 (APOA1) and α−fetoprotein (AFP). Additionally, immunofluorescence analysis was performed to access AFP protein expression.

For ectodermal differentiation, iPSCs were similarly cultured in NS medium, when the cells reached 70%–80% confluency, the NS medium was replaced with the neuronal induction medium (Stemcell Technologies). After 10 days, we observed the morphological changes indicative of successful differentiation, with the cells adopting shapes resembling neuronal cells. These cells were further analyzed for neuronal-specific gene expression, including OLIG2 and MAP2 by qRT-PCR analysis and protein expressions of GFAP and Nestin by immunofluorescence staining.

### Differentiation of iPSCs into iMSCs

For the differentiation of iPSCs into iMSCs, we adhered to the protocol previously developed in our lab [31]. Briefly, the iPSCs were cultured in a 30-mm culture dish with NS medium. When these cells reached 80% confluency, NS medium was replaced with mesenchymal induction medium (STEMdiff-ACF, Stem Cell Technologies) and incubated for 4 days followed by culturing in MesenCult ACF Plus medium for 21 days. By day 21, the cells acquired the matured MSC morphology. This was confirmed through analysis of mRNA and protein expression using qRT-PCR, immunostaining, Western blot and flowcytometry analyses. The characterized cells were further sub-cultured and maintained in MesenCult ACF plus medium.

### Trilineage differentiation potential of iMSCs

To evaluate the trilineage differentiation capacity of the developed iMSCs, the cells were seeded at the density of 7.5 × 10^5^ cells in a 30-mm culture dish containing MesenCult ACF Plus medium. They were subsequently induced to differentiate into chondrocytes, adipocytes, and osteocytes following our established differentiation protocol [31].

To induce osteocyte differentiation, once the iMSCs reached 80% confluency, osteocyte differentiation medium containing Dexamethasone and BMP4 was introduced. After 7 days, the medium was substituted with osteocyte mineralization medium and maintained for 2 weeks. Finally, the induced osteocytes (iOSTs) were evaluated for expression of osteogenic specific markers using qRT-PCR analysis. Alizarin red (ALZ) staining was employed to detect osteocyte mineralization. Later the cells were seeded onto a commercially available collagen-based scaffold to evaluate their adherence to available dental membranes, paving the way for potential clinical applications.

For chondrocytes differentiation, when the iMSCs attained 80% confluency, chondrocyte differentiation medium (Thermo Fisher) was added and cultured for 17 days. The differentiated chondrocytes (iCHON) were characterized by the expression of chondrocyte specific mRNA along with Alcian blue staining.

For adipocyte differentiation, once the iMSCs reached 80% confluency, adipocyte differentiation medium (Thermo Fisher) was introduced. After 11 days, the induced adipocytes (iADIPO) were harvested and characterized through qRT-RCR for adipocyte specific mRNA expression and adipocyte lipid droplets were visualized using Oil red staining.

### Anti-inflammatory assay

To compare the immunomodulatory properties of iMSC and MSC, the cells were treated with 1 μg/mL of LPS for 24 h. The LPS stimulated cells were then harvested and analyzed for the inflammatory and anti-inflammatory marker expression via qRT-PCR following our previously described method [31].

### Cell migration assay

The scratch test is a convenient method for assessing cell migration *in vitro*. The steps include creating an artificial gap by using an insert and seeding a cell monolayer on both sides. The insert is used to provide a homogenous, cell-free space in the middle of attached cells. Then the migration of the cells toward the scratch is quantified by comparing the images at various time points. This migration leads to covering the area and cell to cell contact in the scratch. The major advantage is that it mimics cell migration in tissues and organs [34]. In this study, the cell migration potential of iMSCs and MSCs were assessed and compared by scratch test. iMSC and MSCs were seeded at a concentration of 2 × 10^4^ in the insert placed in 30 mm cell culture plates. After 24 h, the insert was removed (T0) and the percentage of covered area was evaluated after 8 hours (T8) by ImageJ software (NIH).

### Colony formation assay

To determine the capacity of individual iMSCs and MSCs to proliferate and form colonies through clonal expansion, we performed a colony forming assay. This property was assessed by seeding the cells into 30-mm plates at a density of 6.25 × 10^4^ in MesenCult proliferation medium (MesenCult™Proliferation Kit, Stemcell Technologies) for 2 weeks. After a week small-to medium-sized colonies were observed. At the end of 2 weeks culture, the plates were stained by crystal violet. The cells were placed on ice, washed with cold PBS and fixed with 100% ice-cold methanol for 10 min. They were then incubated with 0.05% crystal violet solution in 25% methanol for 20 min. Finally, the cells were washed five times with water, and images were captured using EVOS phase-contrast microscope. The intensity of crystal violet staining was analyzed with ImageJ software.

### Assessment of differentiated osteocytes growth on collagen-based membranes

To evaluate the clinical applicability of iMSC cells, they were differentiated into osteocytes, as described earlier [31]. The osteocytes were then seeded onto a commercial collagen-based membrane (Zimmer BioMend absorbable collagen membrane). The membrane was prepared by cutting it into 8 × 12 mm pieces and inserting in a ring within a 30 mm culture plate. Osteocyte differentiation medium was added to the ring, followed by the addition of 10,000 osteocytes drop wise onto the membrane. After 20 minutes 2 mL of medium was added to the plate, and the cells were incubated for 2 weeks with medium changes every 3 days. After 2 weeks, the membrane was removed and rinsed with PBS. It was then fixed in 2.5% glutaraldehyde for 12 hours at 4°C. The membrane was subsequently sectioned into two pieces to evaluate the osteocyte adhesion and morphology by DAPI staining followed by Scanning Electronic Microscopic analysis. For nuclear staining, the membrane was washed with PBS and DAPI stain solution was added to cover the membrane for 10 minutes. The membrane was washed again, and the images were captured using fluorescent microscope.

### Scanning electron microscopy (SEM)

For SEM analysis, the membrane was first fixed in 2.5% glutaraldehyde in 0.1 M sodium cacodylate buffer (pH 7.4) overnight at 4°C, followed by rinsing with 0.2 M sodium cacodylate buffer (pH 7.4) every 30 min for a total of 1.5 h. Subsequently, the membrane was rinsed with water and subjected to sequential dehydration with increasing concentrations of ethanol (50%, 75%, 95% for 15 min each, and 100% ethanol for 20 min). It was then immersed in hexamethyldisilane (HMDS, Sigma) for 10 minutes and air-dried at room temperature. Before SEM imaging, the membrane was mounted on aluminum stub using carbon adhesive tabs and coated with a gold nanoparticle at ∼100 Å, and then imaged.

### Immunofluorescent staining

We performed immunostaining analysis to quantify the specific proteins within the cells, as described previously [31, 32]. The cells were grown in 4-well chamber slides, washed three times with Dulbecco’s phosphate-buffered saline (DPBS), and then fixed with 4% paraformaldehyde for 5 min. After an additional three washes with DPBS, the cells were permeabilized with 0.1% TritonX-100 in DPBS for 3 min. The cells were then blocked with blocking buffer for 30 min and incubated with primary specific antibodies for overnight at 4°C, followed by a 1-hour incubation with secondary antibodies at 37°C. After washing with DPBS three times, these cells were stained with DAPI, and the staining was analyzed using either a confocal or immunofluorescence microscope.

### Western blot analysis

We conducted Western blot analysis to quantify the proteins of interest as described in our earlier studies [31, 32]. Briefly, the cells were washed with PBS, and lysis buffer was added for protein isolation. The samples were then centrifuged at 12,000 g for 20 min at 4°C. The supernatant was carefully transferred to a new vial and quantified using Bradford’s method with an Accuris™ SmartReader 96‐well microplate absorbance reader at 595 nm. These protein samples were uses for Western blot analysis.

### Software and statistical analysis

To account for any experimental bias, each experiment was conducted in triplicate. The findings are expressed as Mean ± SD. Comparisons were carried out using ANOVA (GraphPad Prism) or the T-test, with a probability value of <0.05 deemed statistically significant. Image analysis was performed using ImageJ software (NIH). Flow cytometry analysis was conducted using FlowJo software.

## Results

### Generation of iPSCs from PDLF: a milestone for dental applications

**FIGURE 1:**
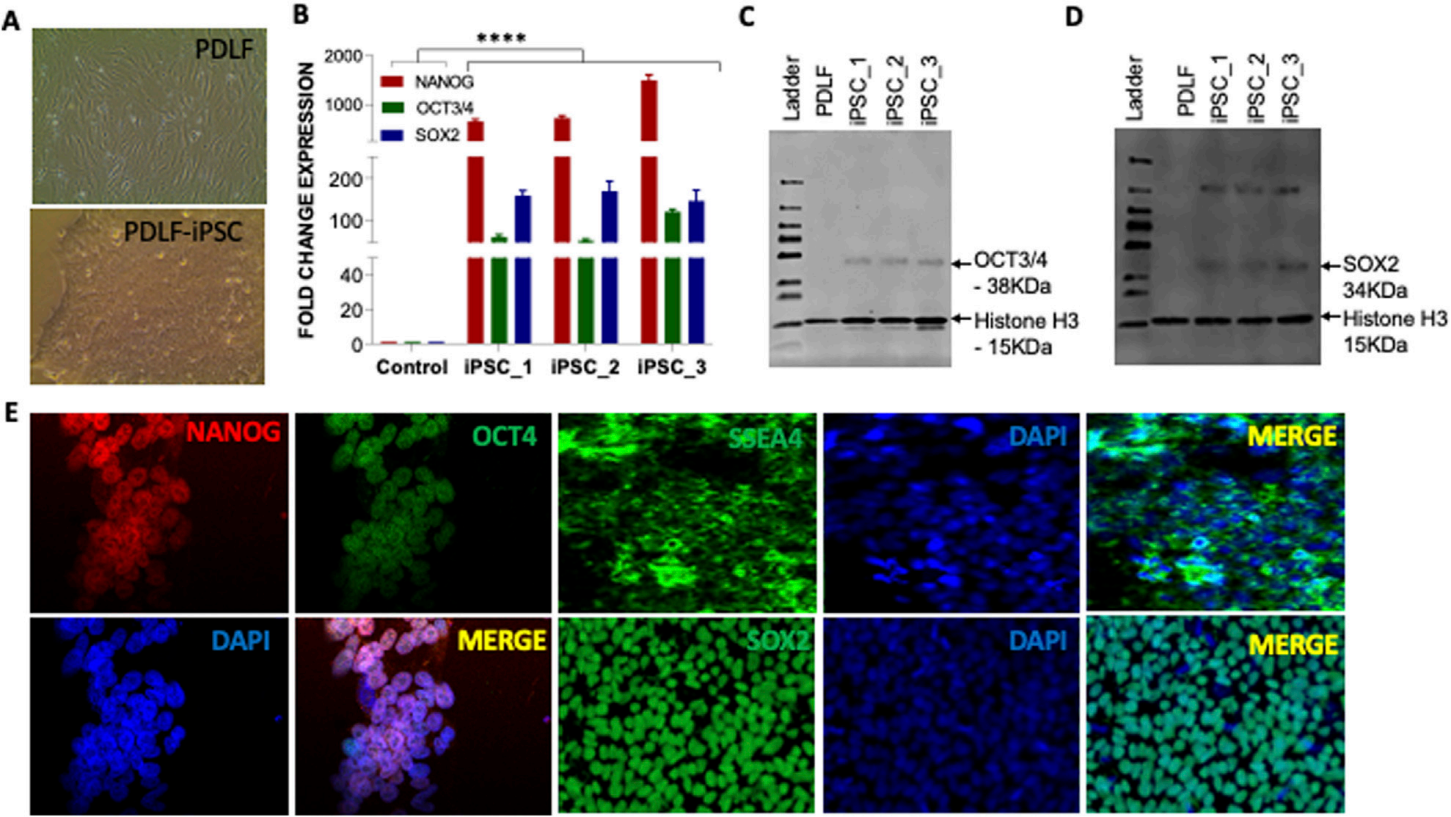
Characterization of the pluripotency of PDLF-iPSCs. **(A)** Microscopic picture of PDLF and PDLF-iPSCs. **(B)** qRT-PCR analysis for the expression of pluripotent genes NANOG, OCT3/4, and SOX2 in three clones of iPSCs on day 11. PDLF cells served as control. The relative mRNA expression was normalized to the 18S gene. Results were expressed as fold change, and the values were calculated as the ratio of induced expression to control expression (****p < 0.00001). **(C)** Western blot analysis showing OCT3/4 and **(D)** SOX2 protein expression levels in iPSC clones derived from PDLF compared to the parent PDLF cells. Histone H3 was used as loading control. **(E)** Pluripotency-specific protein expression, including OCT4, NANOG, SSA4, and SOX2, was assessed via immunofluorescence staining. DAPI staining was performed to visualize the nucleus.

The reprogramming of PDLF into iPSCs was accomplished through mRNA transfection of reprogramming factors combined with a miRNA cocktail, as outlined in our previous articles [32, 33]. During reprogramming, alterations in cell morphology were monitored throughout the process. These changes were sequentially imaged using a phase-contrast microscopy (Supplementary Figure S1). Starting from day 8, several colonies of granulated iPSCs were observed. By the end of day 10, cells with large nuclei that occupied most of the cytoplasm with well-defined round and smooth boarder colony are the typical morphology of pluripotent stem cells also evident (Figure 1A; Supplementary Figure S2). Positive colonies were identified using live TRA1-60 antibody staining and selectively picked and cultured for generating the pure population of PDLF iPSCs. Our findings indicate that iPSCs were successfully generated from PDLF for dental applications.

### Characterization of iPSCs: pluripotency assessment and lineage differentiation

We proceeded with further characterization of the iPSCs by examining the mRNA and protein levels across different clones. Our findings demonstrated a significant elevation in mRNA levels of pluripotent genes OCT3/4, SOX2 and NANOG compared to PDLF (Figure 1B). Moreover, Western blot analysis confirmed notable protein expression of OCT3/4 and SOX2 (Figures 1C, D), while immunofluorescence staining also showed an increased expression of SSEA4, OCT3/4, SOX2 and NANOG markers, respectively (Figure 1E).

**FIGURE 2:**
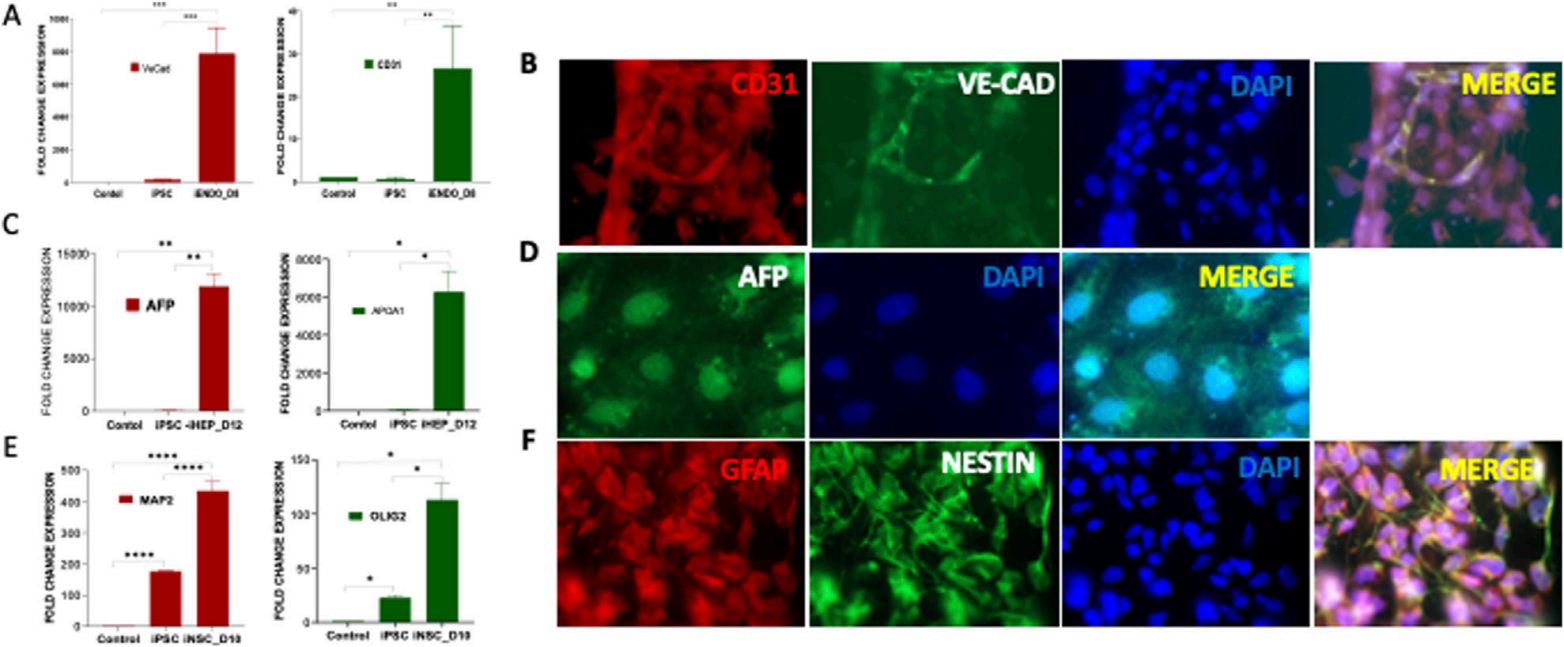
*In vitro* trilineage differentiation of PDLF-iPSCs (iPSCs) **(A)** iPSCs were cultured in mesodermal medium and differentiated to endothelial cells (iENDO). After 8 days (D8) the mRNA expression of VE-Cadherin (VE-Cad) and CD31 was evaluated as an endothelial-specific gene. The significant alterations in the expression of these genes in differentiated cells and control cells (PDLF and iPSCs) (**p < 0.001, ***p < 0.0001) were examined. **(B)** Protein expression of endothelial cell surface markers CD31 (Red), and VE-Cad (Green) were detected by immunofluorescence staining. DAPI (Blue) was used to visualize the nucleus. **(C)** iPSCs were cultured in endodermal differentiated medium and differentiated to Hepatocytes (iHEPO). Hepatocytes specific genes were evaluated for endoderm differentiation. APOA1 and AFP gene expressions were assessed at day12 (D12) (*p < 0.01, **p < 0.001). **(D)** Hepatocyte surface marker AFP (Green) was analyzed using immunofluorescent analysis. **(E)** iPSCs were differentiated to neural cells in neuronal induction medium for 10 days (D10) and the mRNA expression of neuronal specific genes including OLIG2 and MAP2 in the differentiated cells was evaluated (*p < 0.01, ****p < 0.00001). **(F)** Immunofluorescence staining demonstrating the expression of neuronal cell surface markers GFAP (red) and NESTIN (green) in the differentiated cells. Nuclei are counterstained with DAPI (blue). The colocalization of red and green signals indicates the co-expression of GFAP and NESTIN in the cells.

Pluripotency of PDLF-iPSCs were further validated through *in vitro* trilineage differentiation into endothelial cells (mesoderm), hepatocytes (endoderm), and neuronal cells (ectoderm). During endothelial differentiation, qRT-PCR analysis revealed a significant upregulation in mRNA expression of endothelial-specific genes, including VE-Cadherin (VE-CAD) and CD31, compared to the control (PDLF) and iPSCs (Figure 2A). This was further confirmed by immunofluorescence staining, which showed the expression of VE-CAD and CD31 proteins (Figure 2B). Following endodermal differentiation, the cells exhibited elevated mRNA levels of hepatocyte-specific markers, including α-fetoprotein (AFP) and Apolipoprotein A1 (APO1) (Figure 2C). Immunostaining further confirmed strong AFP protein expression (Figure 2D), affirming their successful differentiation into the endodermal lineage. During neuronal differentiation, the differentiated cells showed enhanced expression patterns of neuronal specific genes, including oligodendrocyte transcription factor 2 (OLIG2) and microtubule associated protein 2 (MAP2) than the iPSCs and PDLF cells (Figure 2E). Moreover, the expression of Nestin and GFAP in the differentiated cells was evident in through immunofluorescence staining (Figure 2F). Overall, these findings affirm the pluripotent nature of the reprogrammed iPSCs, demonstrating their ability to differentiate into all three germ layers of the embryo.

### Comprehensive characterization and differentiation potential of iPSCs into iMSCs

**FIGURE 3:**
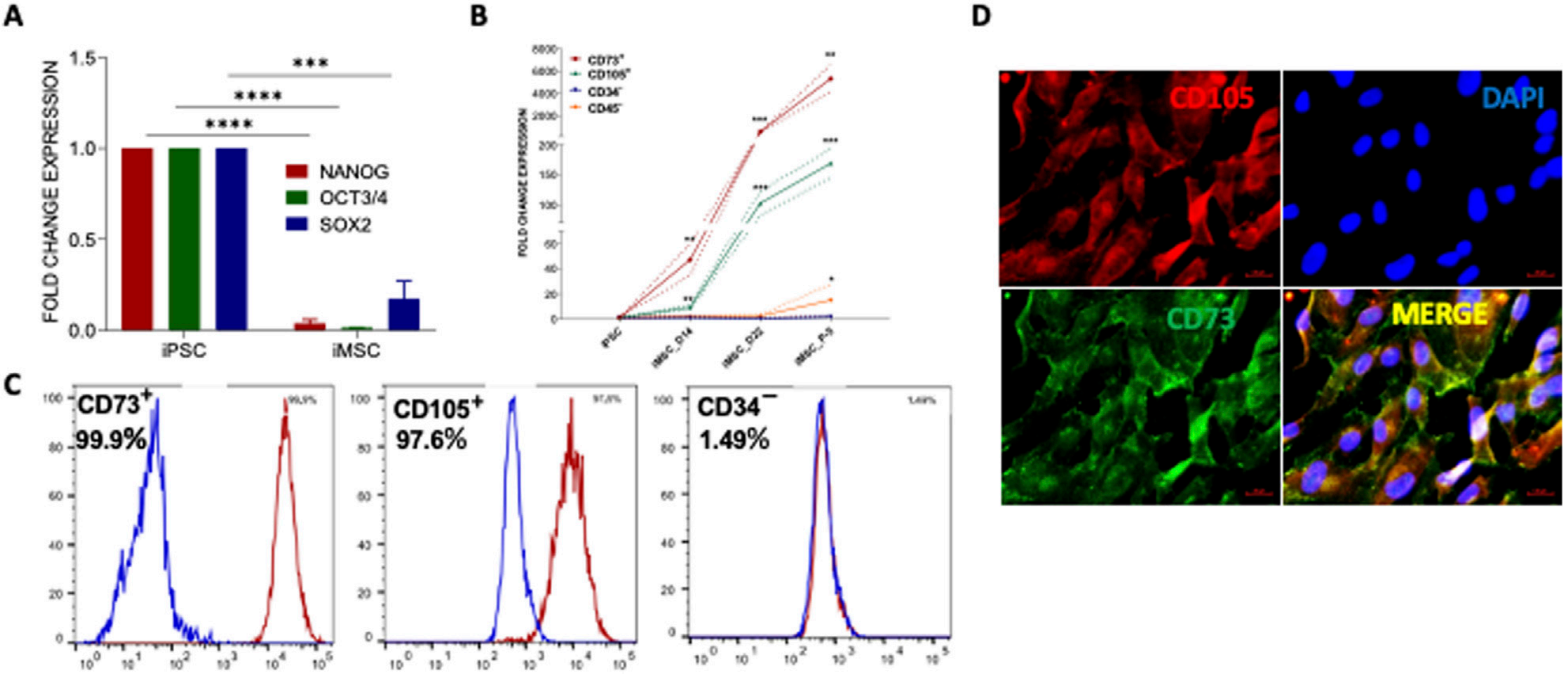
Generation and characterization of PDLF-iMSCs. **(A)** The expression of iPSC markers OCT4, NANOG and SOX2 was significantly decreased in the differentiated cells (PDLF-iMSCs). The mRNA expression levels were normalized to 18s rRNA. **(B)** Expression analysis of mesenchymal-specific genes and hematopoietic markers in PDLF-iMSCs. The differentiated cells (PDLF-iMSCs) showed a significant increase in the expression of mesenchymal-specific genes CD73 and CD105 at different time points compared to iPSCs (**p < 0.001, ***p < 0.0001). In contrast, the expression of hematopoietic markers CD34 and CD45 was negligible or significantly lower in PDLF-iMSCs compared to iPSCs (*p < 0.01). **(C)** Flow cytometric analysis revealing over 95% cell expression of mesenchymal surface markers CD73 and CD105, alongside non-expression of CD34. Isotype control antibodies served as negative controls to validate specificity. **(D)** The Protein expression of mesenchymal markers, CD105 and CD73 in iMSCs was significant and confirmed by immunofluorescence staining.

After confirming the pluripotent nature of PDLF-derived iPSCs, they were further differentiated into iMSCs through our standard lab protocol as described earlier [31]. Morphological changes during the differentiation process were observed and documented (Supplementary Figure S3). To fully access the differentiation of iPSCs to iMSCs, we examined the expression of iPSC-specific genes, including OCT3/4, Nanog, and SOX2. Meanwhile, the iMSC clones displayed decreased expression pattern of pluripotent genes indicating the loss of embryonic properties during differentiation (Figure 3A). To characterize the resulting iMSCs, qRT-PCR analysis was performed for specific mesenchymal genes, including CD73 and CD105 as positive markers and CD34 and CD45 as negative markers. The results indicated an elevated expression of positive markers along with a lower expression of negative markers (Figure 3B). Interestingly, we found that the iMSCs exhibited a greater level of maturation, with heightened expression of MSC markers in comparison to iPSCs. The protein expression of some of these markers was confirmed by flow cytometric analysis, and immunostaining. Flowcytometric analysis demonstrated that over 95% of the cells expressed the mesenchymal cell surface markers CD73 and CD105. Conversely, the expression of the negative marker CD34 was lowered (Figure 3C). This was corroborated by the high protein expression levels of CD73 and CD105 markers in immunofluorescent staining (Figure 3D).

### Trilineage differentiation potential of iMSCs into osteocytes, chondrocytes, and adipocytes

**FIGURE 4:**
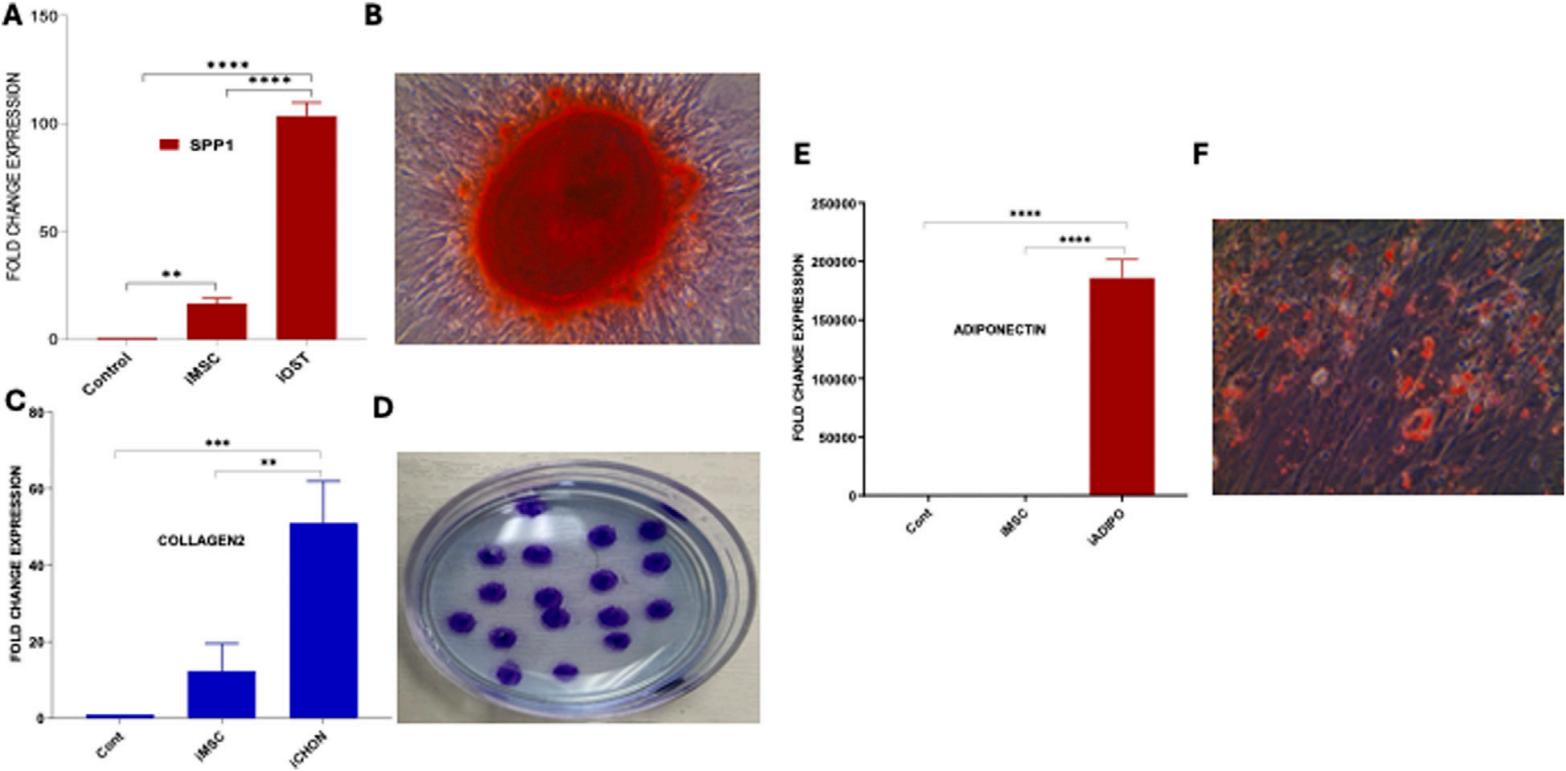
Trilineage differentiation of iMSCs. **(A)** Comparative analysis of SSP1 expression in iOSTs, iMSCs, and control cells. iOSTs exhibited significantly higher expression of the osteocyte-specific gene SSP1 compared to iMSCs and control cells (****p < 0.00001). **(B)** The calcium depositions of these cells were prominently stained by Alizarin Red. **(C)** The expression of the chondrocyte-specific marker collagen-2 was significantly higher in induced chondrocyte differentiated cells (iCHON) compared to iMSCs and control cells (**p < 0.001, ***p < 0.0001). **(D)** Alcian blue staining of iCHON cell colonies, revealing several stained areas on the culture plate, indicative of chondrocyte-specific matrix production. **(E)** In adipocyte differentiation, the induced adipocyte (iADIPO) cells showed significantly higher expression of the adiponectin gene compared to iMSCs and control cells (****p < 0.00001). **(F)** Induced adipocyte (iADIPO) cells positively stained with Oil Red O, indicating the presence of lipid droplets characteristic of adipocytes.

Furthermore, these iMSCs were evaluated for their multipotent differentiation potential into induced osteocytes (iOSTs), induced adipocytes (iADIPO), and induced chondrocytes (iCHON). For osteocyte differentiation, iMSCs were cultured in iMSC medium and then replaced with osteocyte differentiation medium. Subsequently, they were cultured in osteocyte mineralization medium for 2 weeks. The morphological changes of the cells during the osteocyte differentiation process were observed under phase-contrast microscopy (Supplementary Figure S4). The qRT-PCR analysis showed the mRNA expression of the osteocyte-specific gene SSP1 was significantly increased in the iOST (Figure 4A). Furthermore, the presence of calcium deposits in the iOSTs was clearly highlighted by Alizarin Red staining, indicating their role in bone mineralization and calcium regulation, which are essential for bone strength and structure (Figure 4B). The iMSCs were further differentiated into iCHONs. The differentiated iCHONs showed significantly increased expression levels of the chondrocyte-specific marker collagen-2 compared to the undifferentiated iMSC control (Figure 4C). Multiple stained areas were visible on the cultured plate when subjected to alcian blue staining, which specifically highlights chondrocytes (Figure 4D). The iMSCs also demonstrated their ability to differentiate into adipocytes. The resulting adipocytes displayed high expression of the adipocyte-specific marker adiponectin, confirming pure clones (Figure 4E). Furthermore, the deposition of fat and lipid droplets in iADIPO cells was confirmed by oil red O staining (Figure 4F). Overall, our data clearly indicate that iMSCs possess markedly superior multipotential differentiation abilities, similar to UC-MSCs, highlighting their greater potential for diverse clinical applications.

### Comparative analysis of anti-inflammatory characteristics of iMSCs with standard MSCs

**FIGURE 5:**
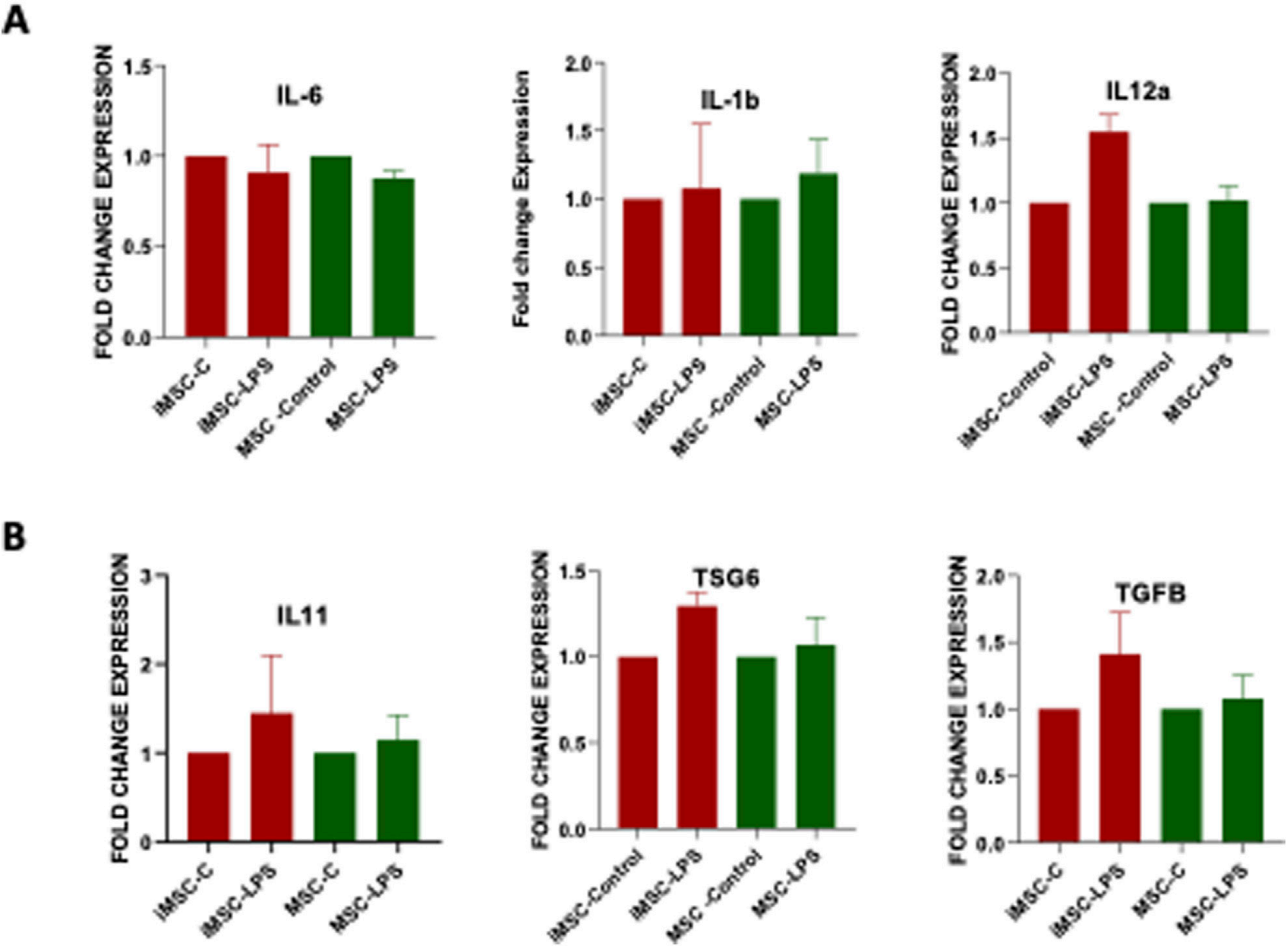
Comparative analysis of **(A)** pro-inflammatory (IL-1β, IL-6, IL-12α) and **(B)** anti-inflammatory (IL-11, TGF-β, TSG-6) gene expression in PDLF-iMSCs and UCMSCs after 24 h of LPS stimulation, showing no significant differences in mRNA expression levels between the two cell types.

The anti- inflammatory cytokines play a crucial role in regulating the immune response and counteracting the effects of pro-inflammatory cytokines [35]. In the present study, we aimed to evaluate the anti-inflammatory potential of iMSCs by comparing them to MSCs as the control group. To assess the anti-inflammatory properties, both iMSCs and MSCs were treated with lipopolysaccharides (LPS-Sigma-Aldrich). After 24 h, we examined the gene expression levels of major anti‐inflammatory cytokines such as IL‐11, TGF‐β and TSG‐6, as well as pro‐inflammatory cytokines such as IL‐6, and IL1β, using qRT-PCR analysis. The results indicated that there was no significant difference in the expression of these cytokines between iMSCs and MSCs. Both cell types exhibited similar gene expression patterns in response to the inflammatory stimulus. Importantly, iMSCs showed a slightly higher expression of IL-11, TGFα, and TSG6, which are known to be anti-inflammatory markers (Figure 5A). On the other hand, the expression of pro-inflammatory markers, including IL-1β, IL-6, and IL-12α was relatively lower in iMSCs compared to MSCs, although these differences were not statistically significant (Figure 5B). The proinflammatory gene expression is lower because of anti-inflammatory nature of iMSCs. These findings suggest that iMSCs possess comparable anti-inflammatory properties to MSCs, as evidenced by their ability to express anti-inflammatory cytokines in response to an inflammatory stimulus. While there were subtle differences in the expression levels of certain cytokines, overall, iMSCs demonstrated similar immunoregulatory capabilities to MSCs in the context of inflammation.

### Enhanced migratory and proliferative abilities of iMSCs compared to MSCs

**FIGURE 6:**
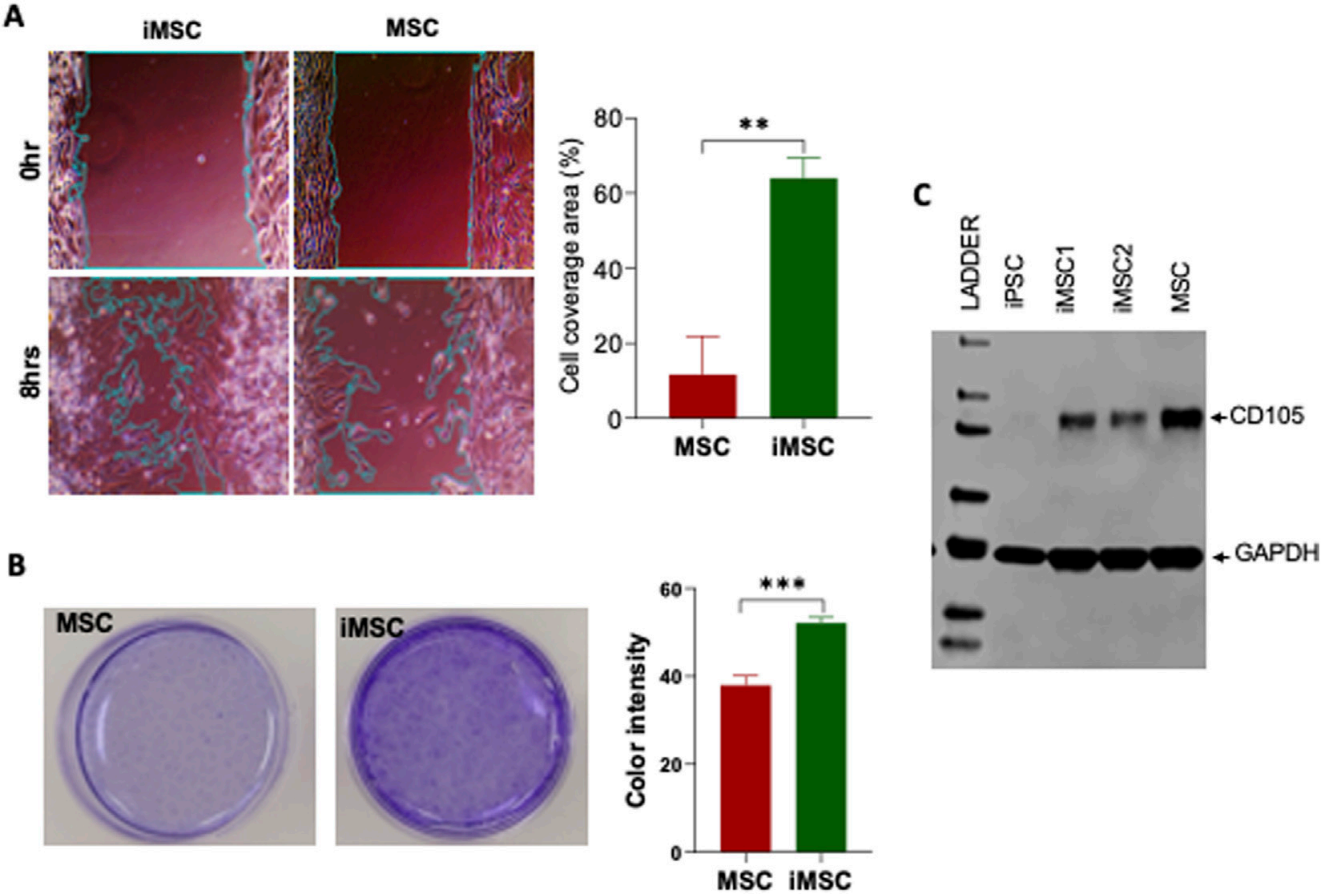
The migration and colony formation potential of PDLF-iMSCs compared to UCMSCs. **(A)** Scratch assay showing cell migration after 8 h. The covered area in the PDLF-iMSCs is significantly larger than in the UCMSCs (**p < 0.001). **(B)** Colony formation of PDLF-iMSCs and UCMSCs after 2 weeks. PDLF-iMSCs formed more colonies with increased blue staining intensity compared to UCMSCs (***p < 0.0001). **(C)** Western blot analysis demonstrating comparable CD105 (71 KDa) protein expression in PDLF-iMSCs and MSCs. GAPDA (36 KDa) protein serves as a loading control.

Scratch assay, an *in vitro* model of cell migration, was used to assess the migratory capacity of iMSCs compared to MSC. The results revealed that iMSCs covered approximately 60% of the scratch area, demonstrating a significantly higher migratory capacity (three times greater than that of MSCs) 8 hours after the scratch was made (Figure 6A). These findings highlight the superior migration ability of iMSCs in terms of covering the scratch area. To evaluate cellular survival, differentiation ability, and growth potential, a clonogenic assay was performed on iMSCs and MSCs. This assay is an *in vitro* method that measures how effectively a single cell can proliferate into a substantial colony through clonal expansion. The results demonstrated that iMSCs were capable of significantly forming more colonies compared to MSCs (Figure 6B). This indicates that iMSCs have a higher proliferation capacity than MSCs. Apart from migratory and proliferative characteristics, iMSCs also exhibited CD105 protein expression similar to MSCs, as demonstrated through western blot analysis (Figure 6C). These properties position iMSCs as an alternative autologous source and a favorable option for clinical applications compared to MSCs.

### iMSCs for regenerative dentistry: applications on collagen membranes

**FIGURE 7:**
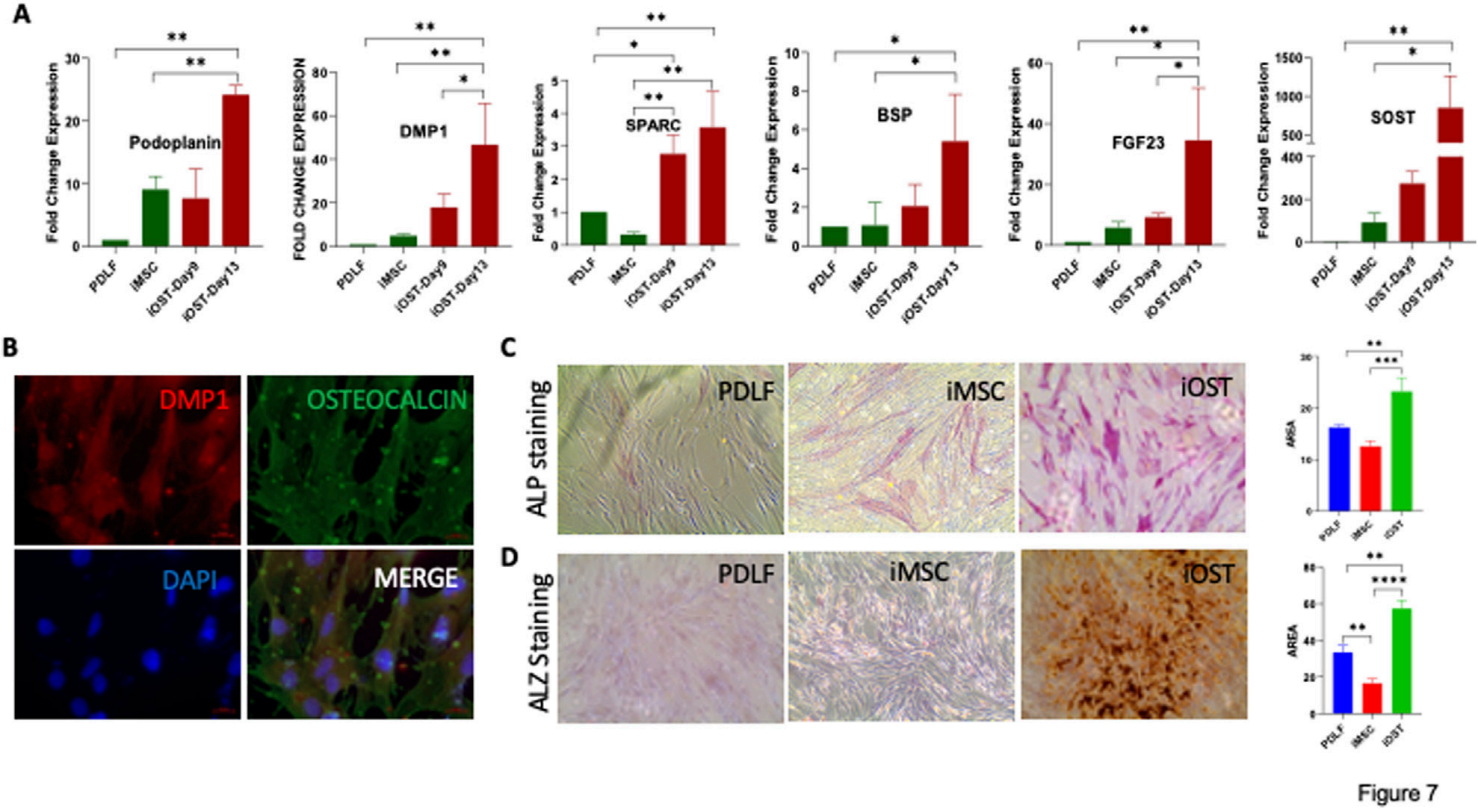
Osteocyte differentiation potential of iMSCs. **(A)** Expression levels of osteocyte-specific genes in PDL-iOSTs at days 9 and 13, compared to PDLF and PDLF-iMSCs. Significant increases in gene expression in PDL-iOSTs over time are indicated (*p < 0.01, **p < 0.001, ***p < 0.0001, ****p < 0.00001). **(B)** Immunofluorescence analysis of PDLF-iOSTs demonstrating the expression of DMP1 (red) and osteocalcin (green) proteins. Mineralization and calcium deposition in PDLF-iOSTs detected by **(C)** ALP and **(D)** Alizarin Red (ALZ) staining. The intensity of staining, indicative of mineralization, was measured using ImageJ software to assess the area of staining.

For regenerative dentistry applications, we programmed our iMSCs to differentiate into terminally matured osteocytes. The mRNA expression levels of early marker Podoplanin, mineralizing marker DMP1, and mature or late markers associated with osteocyte differentiation namely BSP, FGF23, SOST, and SPARC were notably elevated in these differentiated cells (Figure 7A). Furthermore, these cells were positively stained with fluorescent antibodies for osteocalcin and DMP1, confirming their osteocyte-like characteristics (Figure 7B). APL staining and Alizarin Red staining revealed prominent calcium deposits in the iOSTs (Figures 7C, D). These findings collectively manifest that PDLF-iOSTs closely replicate the properties of human osteocytes.

**FIGURE 8:**
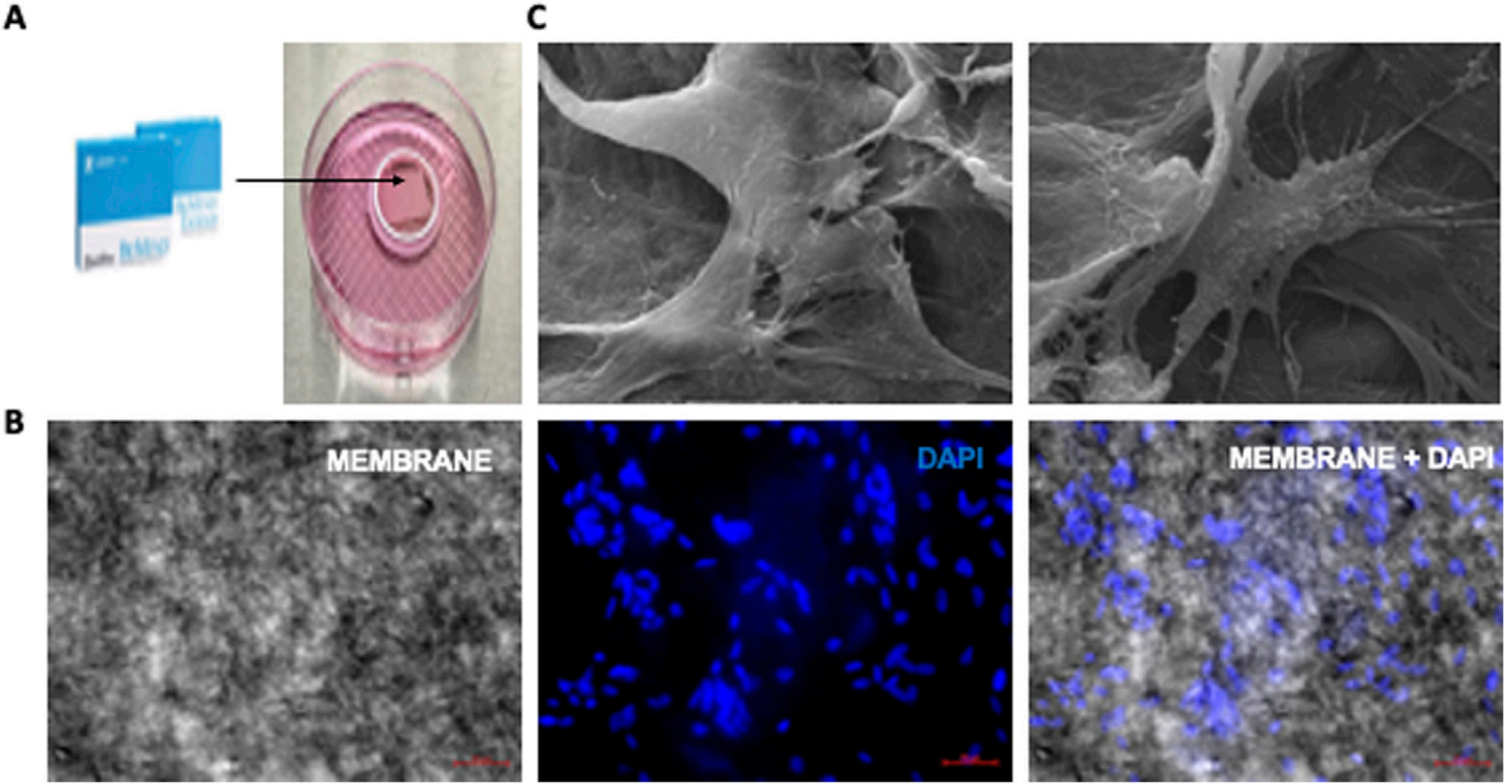
Attachment of PDLF-iOSTs to a commercial collagen-based membrane. **(A)** A piece of collagen membrane placed in a ring-shaped instrument. **(B)** DAPI staining and fluorescent imaging of PDLF-iOSTs attachment on a collagen-based membrane. The left image shows the membrane alone, the center image highlights the nuclei of attached and proliferated cells, and the right image displays the membrane with attached cells. A substantial number of cells remained on the membrane following the preparation process. **(C)** SEM images of PDLF-iOSTs adhered to the collagen-based membrane through their extended processes.

Subsequently, we transferred these cells PDLF-iOSTs onto a collagen membrane (Figure 8A). After fixation and preparation, cell adhesion was assessed using DAPI staining via immunofluorescence microscopy and the ultrastructure was examined with scanning electron microscopy (SEM). DAPI staining displayed a substantial number of cells attached to the membrane (Figure 8B), and SEM images provided insights into the cell morphology and adhesion (Figure 8C). Overall, these findings illustrate that PDLF-iOSTs successfully adhered to the collagen membrane, underscoring their promising potential for applications in regenerative medicine and tissue engineering.

## Discussion

Regenerative dentistry has emerged as a promising approach for repairing and replacing lost oral tissues and organs. Advances in molecular biology and the understanding of tissue development genes have paved the way for developing functional and biocompatible oral tissues. Teeth and their supporting tissues, such as PDLF, are easily accessible source of stem cells, making them highly relevant for personalized regenerative therapies [36, 37]. The PDLF cells can be conveniently isolated during routine procedures, such as tooth extractions, third molar removal, makes them a compelling option for regenerative treatments, particularly in the field of personalized dentistry [38]. Studies have shown that MSCs are the most used cell type in regenerative dentistry [39]. However, MSCs face challenges such as limited survival and proliferation in harsh environments [40–42], immune rejection risks [43], donor age-related cell senescence [44] and oncogenic potential due to prolonged *in vitro* culture [45].

To address these challenges, our study utilized a novel, viral- free reprogramming strategy [31] combining messenger RNA (mRNA) and microRNA (miRNA), to generate iPSCs from PDLF cells. This method eliminates the safety concerns associated with viral vectors and resulted in iPSCs with robust regenerative properties and trilineage differentiation potential, a crucial factor for their therapeutic use. The PDLF-derived iMSCs demonstrated strong differentiation capacities into osteocyte, adipocyte, and chondrocyte, comparable to other sources of MSCs but with the advantage of a minimally invasive collection process. These findings align with previous research on urinary epithelial-derived iMSCs, further validating the regenerative capabilities of iMSCs from various sources [31].

iMSCs have demonstrated superior immune suppression compared to MSCs, as supported by previous studies [46]. To further explore this, we subjected both iMSCs and MSCs to LPS to assess their inflammatory properties. Our findings revealed that the iMSCs showed mild inflammatory and high anti-inflammatory responses when exposed to LPS which is comparable to those of MSCs, indicating that iMSCs maintain immune regulatory functions. LPS is indeed a potent stimulator of immune cells; however, in the context of iMSCs, it exhibits a more moderate stimulatory effect. iMSCs are known for their anti-inflammatory properties, which could mitigate the typical proinflammatory response to LPS. This suggests that iMSCs retain the immune-regulatory functions typical of iMSCs, making them a promising candidate for treating inflammatory conditions, not only in dental applications but also in broader clinical settings.

In our study, iMSC-derived osteocytes successfully adhered to commercially available collagen-based resorbable membranes, demonstrating their suitability for bone regeneration and guided tissue repair. The ability of iOSTs to adhere to a commercial collagen membrane is significant, as it demonstrates that these cells can attach, migrate, and potentially proliferate, key characteristics for their application in tissue engineering, particularly in bone regeneration and wound healing. The chemically crosslinked collagen membrane used in this study effectively supports guided bone regeneration by providing a scaffold conducive to cell attachment and growth. Additionally, its slower degradation rate helps maintain the structural integrity and stability of the injury site, enhancing effective healing while minimizing the risk of deformities [47]. Importantly, the use of iOSTs on such membranes can serve a dual purpose: aiding in bone regeneration prior to or during dental implant placement and creating a favorable environment for successful guided bone regeneration surgery. This approach holds promise for addressing gaps in current regenerative strategies.

Our findings demonstrate that the iMSCs derived from PDLF not only possess superior regenerative properties but also offer a more accessible and cost-effective cell source for regenerative therapies. The advantage of PDLF derived iMSCs is that PDLFs will have periodontal niche which will elucidate better periodontal regeneration and craniomaxillofacial regeneration and they also have low immunogenicity compared to blood and skin cells [48]. Moreover, the use of iMSCs derived from dental tissues for clinical applications, such as bone regeneration and soft tissue repair, could significantly improve the outcomes of regenerative dental procedures, such as sinus lifts, bone augmentation, and guided tissue regeneration. Our study also highlights the advantages of iMSCs compared to umbilical cord-derived MSCs, which are widely stored and used in clinical trials. In many developing countries, there is a growing trend of storing umbilical cord-derived MSCs at the time of delivery, resulting in significant financial gains for stem cell storage companies. Through this research, we aim to raise awareness that iMSCs can be generated from adult body cells, such as PDLF, can provide an equally effective or even potentially superior to MSCs. This comparison underscores the feasibility of utilizing easily accessible body-derived cells for therapeutic purposes. While we acknowledge the limitation of donor variability and the small sample size, the intent of this study is to explore this alternative approach and establish a basis for further, more extensive research in the future.

In conclusion, PDLF-derived iMSCs offer a cost-effective, accessible, and viable cell source with superior regenerative properties for dental and craniofacial applications. Further studies should focus on refining these techniques, addressing and explore their clinical application in a broader range of therapeutic contexts.

## Data Availability

The original contributions presented in the study are included in the article/Supplementary Material, further inquiries can be directed to the corresponding author.
