# Supplementary material for: Induced mesenchymal stem cells generated from periodontal ligament fibroblast for regenerative therapy
Source: Exp Biol Med (Maywood). 2025 Feb 3;250:10342. doi: 10.3389/ebm.2025.10342 (PMC11830513; doi:10.3389/ebm.2025.10342)
Supplement: Supplementary file 1 [file DataSheet1.PDF]

ONLINE SUPPLEMENTARY MATERIALS

Supplemental Figure S1. Non-Viral Reprogramming of Human Periodontal Ligament Fibroblasts into Induced Pluripotent Stem Cells

A-F, Morphological changes during the non-viral reprogramming of PDLF cells into iPSCs were documented using phase contrast microscopy at various time points: day 1, day 3, day 6, day 8, day 10, and day 13. Starting from day 10, several granulated colonies resembling human embryonic stem cell colonies were observed. The images illustrate the progression of reprogramming and the emergence of iPSC-like colonies.

Supplemental Figure S2. iPSCs colony having round and smooth boarder morphology

A-D, Whole iPSC colony to demonstrate good round morphology and have a smooth border. **A,** the iPSC colony stained with immunofluorescence Nanog antibody. **B,** iPSC colony stained with immunofluorescence SSEA4 antibody. **C,** merged immunofluorescence image of Nanog and SSEA4. **D,** Phase-contrast microscopic image of round and smooth boarder iPSC colony.

Supplemental Figure S3. Conversion of PDLF-Derived iPSCs into iMSCs

A-F, Sequential differentiation process of PDLF derived iPSCs into iMSCs was monitored over 21 days. Morphological changes were captured using phase contrast microscopy at various stages: day 1, day 5, day 14, day 16, day 18, and day 23. These images illustrate the transformation of iPSCs into iMSCs, highlighting key morphological changes at each documented time point.

Supplemental Figure S4. Derivation of induced osteocytes (iOSTs) from iMSCs

A-F, Phase contrast microscopy images illustrating sequential morphological changes during the transition from iMSC to iOST at various time points including day1, day3, day6, day9, day11 and day13.

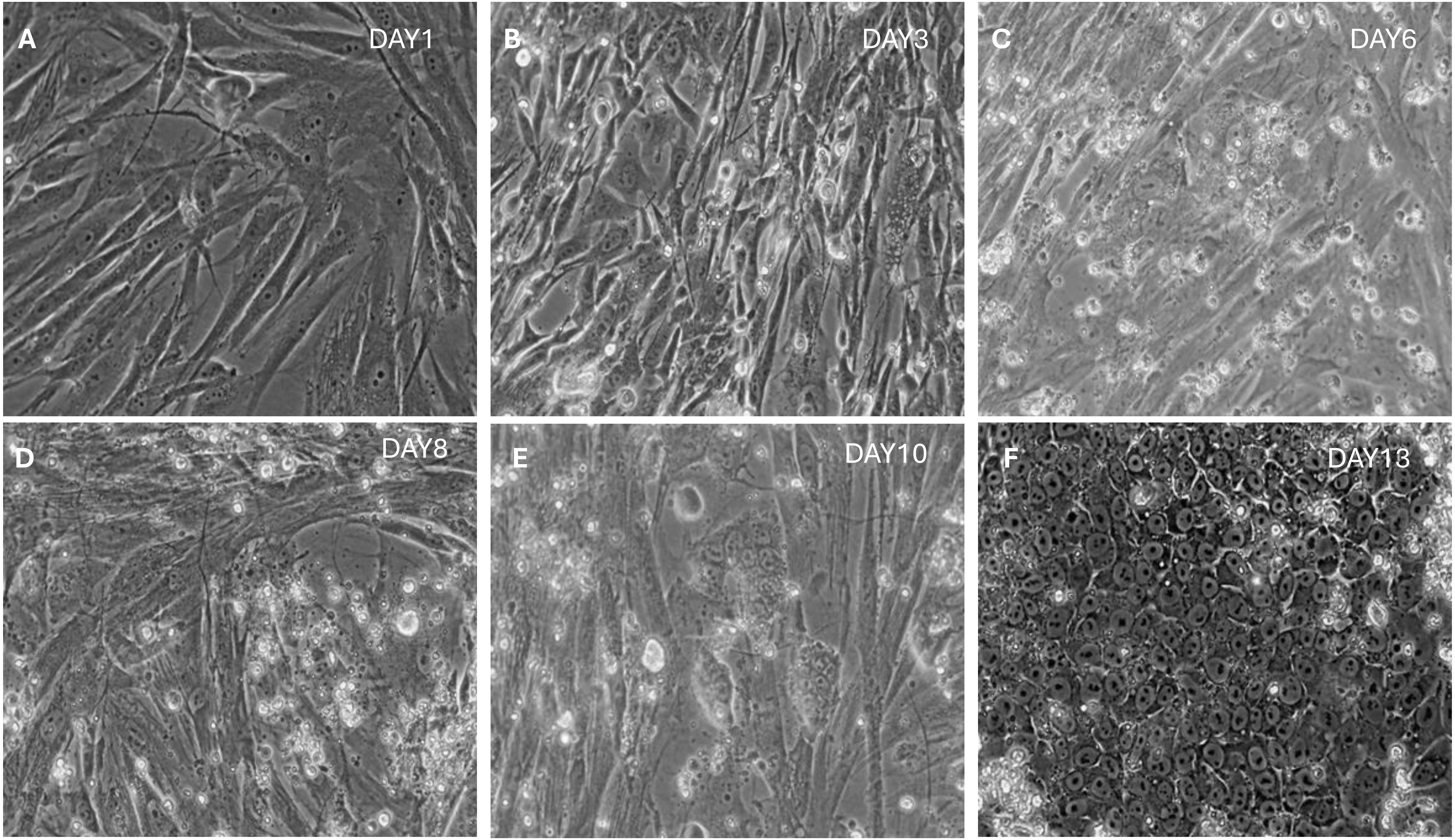

Sequential changes of PDLF into iPSCs during reprogramming

Supplement Figure 1

40X

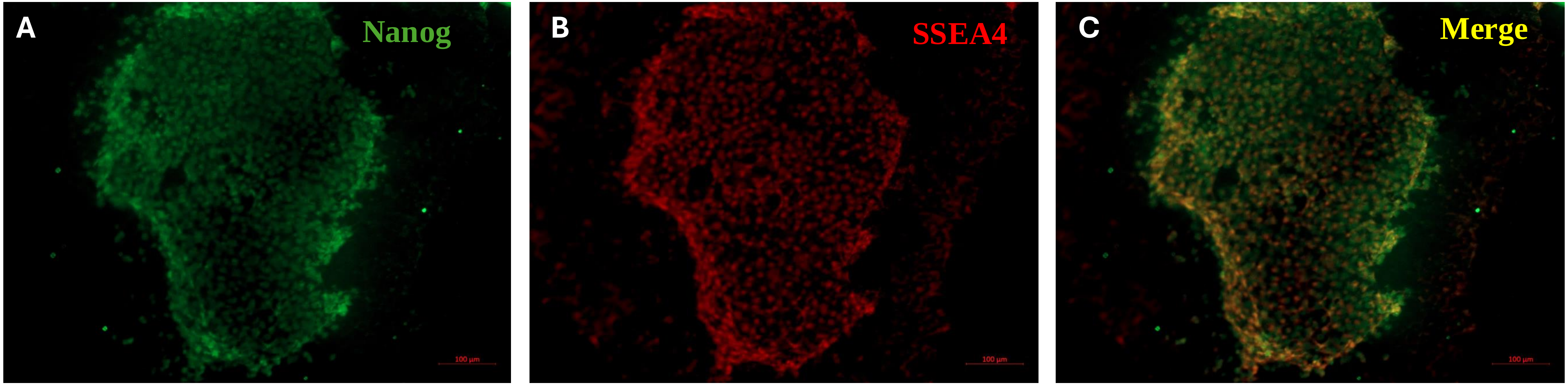

Phase contrast microscopic image

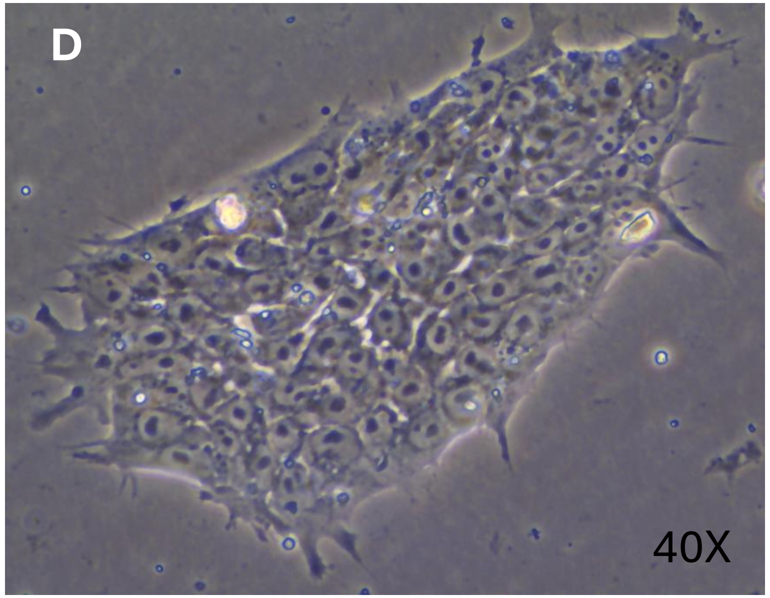

Supplement Figure 2

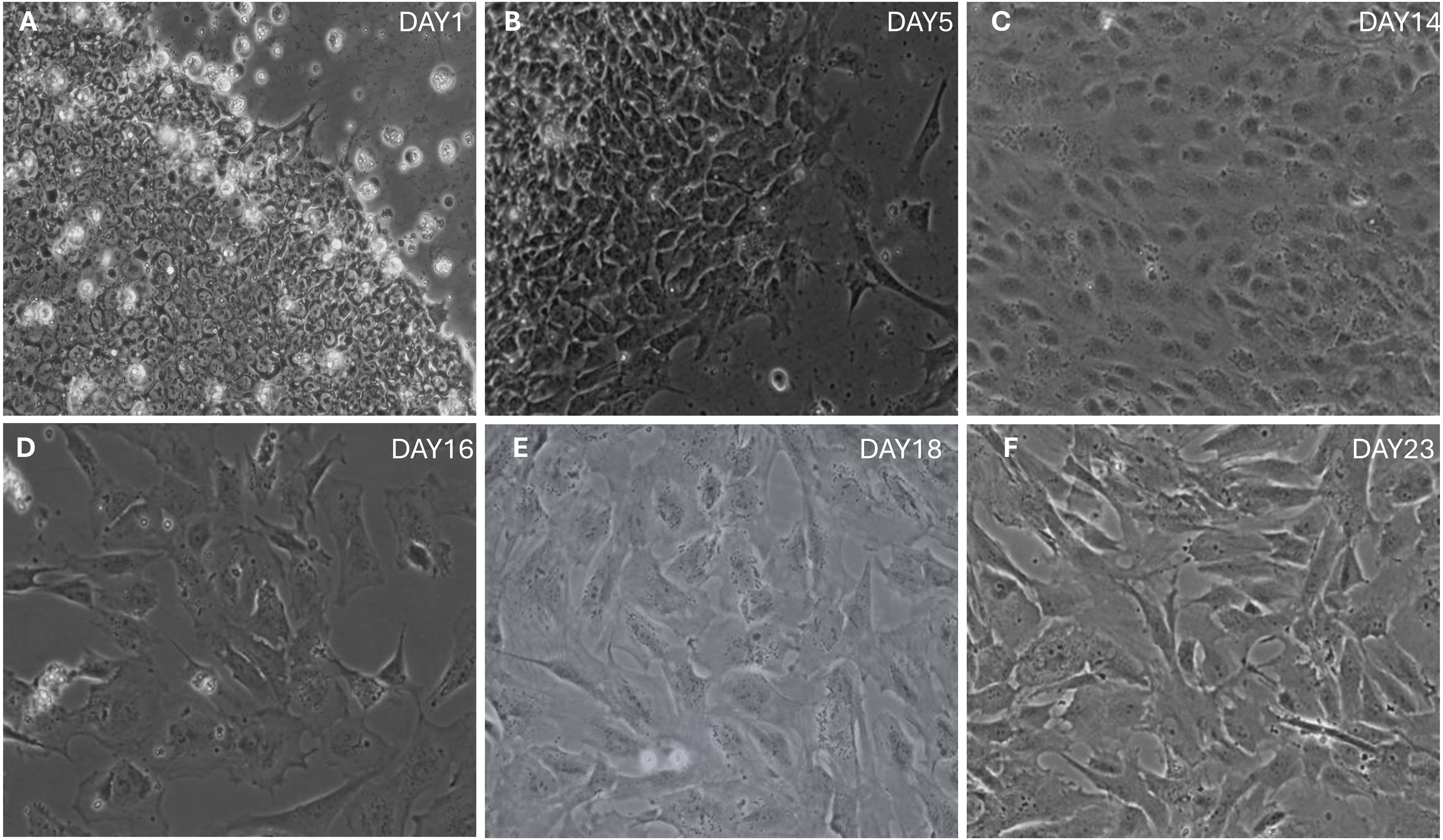

Supplement Figure 3

40X

Sequential changes during differentiation of iPSCs into iMSCs

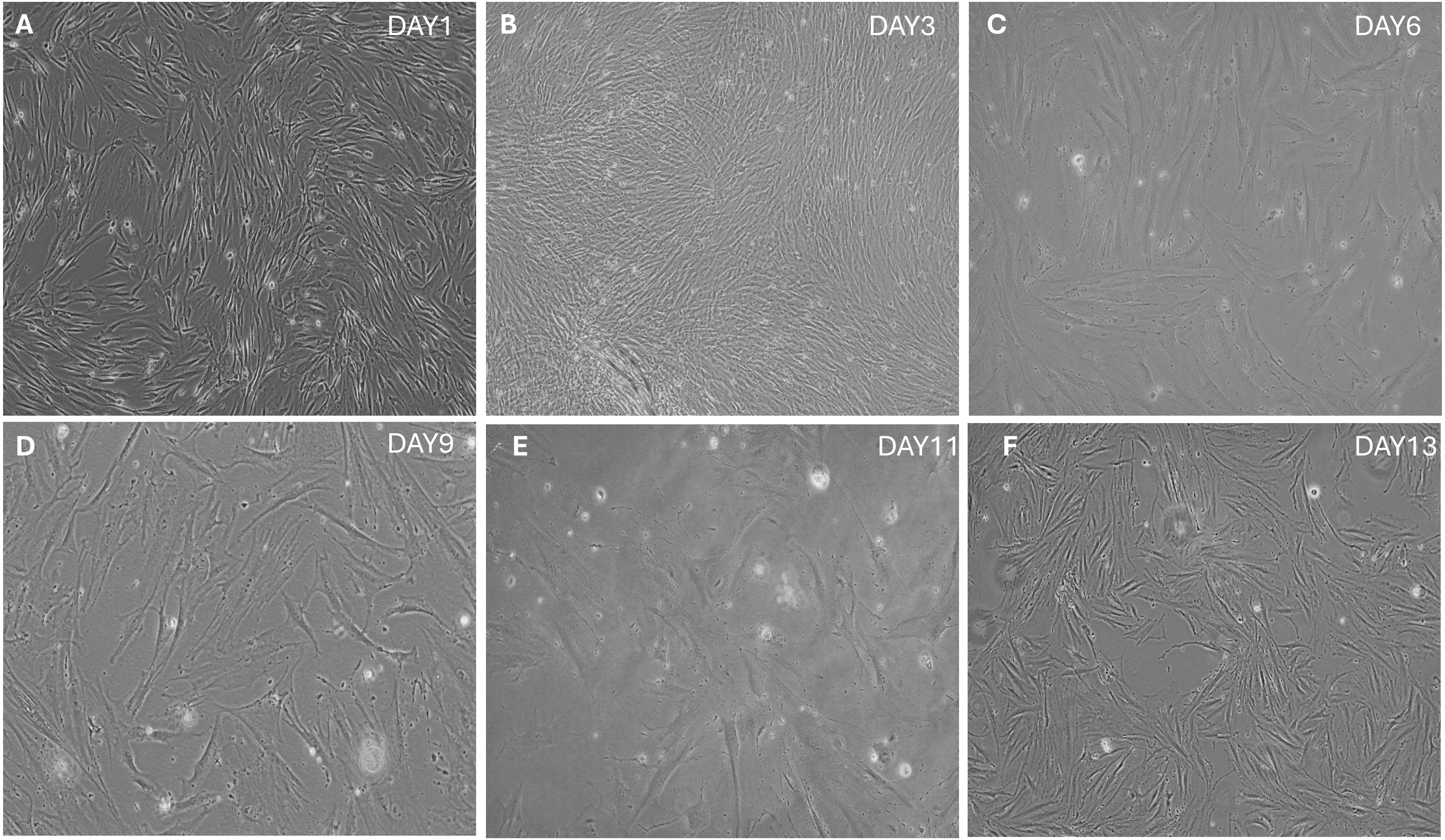

Differentiation of PDLF-iMSCs into -iOST

Supplement Figure 4 10X
